# Cracking Chordoma's Conundrum: Immune Checkpoints Provide a Potential Modality

**DOI:** 10.7150/ijms.109721

**Published:** 2025-04-22

**Authors:** Weihai Liu, Moksada Regmi, Xiaodong Chen, Shikun Liu, Ying Xiong, Yuwei Dai, Yingjie Wang, Jun Yang, Chenlong Yang

**Affiliations:** 1 State Key Laboratory of Vascular Homeostasis and Remodeling, Department of Neurosurgery, Peking University Third Hospital, Peking University, Beijing 100191, China.; 2 Center for Precision Neurosurgery and Oncology of Peking University Health Science Center, Peking University, Beijing 100191, China.; 3 Peking University Health Science Center, Beijing 100191, China.

**Keywords:** chordoma, differential expression, immune checkpoints, immune checkpoint inhibitors

## Abstract

**Objectives:** Chordoma, a rare malignant tumor, is notably resistant to conventional treatments including chemotherapy, radiotherapy, and targeted approaches. Immunotherapy, successful in treating other cancer types, presents a promising avenue. However, the immune microenvironment of chordoma is poorly understood, highlighting the need to investigate immune checkpoints and their potential as therapeutic targets in this context.

**Methods:** We performed an integrated analysis of chordoma using public datasets (GSE224776, GSE56183, GSE239531) and our RNA-seq data (11 samples). Differential expression analysis (limma), gene set enrichment analysis (GSEA, clusterProfiler), immune cell infiltration assessment (ESTIMATE, immunedeconv), weighted gene co-expression network analysis (WGCNA), consensus clustering, and machine learning were employed to identify key immune-related gene modules, immunogenic subtypes, and central immune regulators.

**Results:** Hierarchical clustering and principal component analysis segregated chordoma from control samples post quality control. Differential expression analysis identified 2825 upregulated and 1693 downregulated genes, with significant upregulation of immune checkpoints, including PD-1 and CTLA-4. GSEA highlighted enhanced immune-related processes, particularly inflammatory responses, antigen presentation, and immune cell activation. Immune cell deconvolution demonstrated selective enrichment of memory T cells and macrophages, alongside downregulation of neutrophils and decreased effector cell scores. Consensus clustering identified a highly immunogenic chordoma subtype (Cluster 1), and WGCNA and machine learning converged on CCR7 as a central immune regulator, with core T cell-associated genes correlating with immune cell distribution patterns.

**Conclusion:** This study characterizes the chordoma immune landscape, highlighting elevated immune checkpoints, distinct immunogenic subtypes, and a T cell-centered regulatory network. These findings support immune checkpoint inhibitors and other immunotherapies as promising treatments.

## Introduction

Chordoma is a rare, aggressive bone tumor from notochord remnants, affecting about 0.1 per 100,000 people, and poses major treatment challenges 1, 2. These tumors predominantly occur in the sacrococcygeal region and skull base, often leading to debilitating compression of adjacent neurovascular structures 3. Despite being rare, chordomas are notorious for their aggressive nature, high recurrence rates, and resistance to conventional treatments, including radical resection and high-dose irradiation, resulting in substantial morbidity and mortality 4.

Surgical intervention remains the primary treatment for chordoma, offering benefits in long-term survival and local control. However, the tumor's close proximity to critical neural structures complicates complete removal. In many cases, surgeons are unable to excise the entire tumor, and around 30% of patients experience distant spread, necessitating additional surgeries that may not always be effective 5, 6. Even with macroscopic complete resections, more than 50% of patients face locoregional recurrence, with five-year survival rates typically ranging from 65% to 70% 7-11. Unfortunately, surgery can also cause severe pain, loss of function, and, in some cases, death 12.

Radiotherapy, while offering local control and providing therapeutic and palliative benefits, is limited in dosage escalation due to the proximity to brainstem and spinal cord. Patients with recurrent or metastatic chordoma face limited systemic therapy options, and the prognosis remains dismal with a median overall survival of 6-9 months 13, 14. Chordoma is resistant to chemotherapy, potentially due to a low proliferation pool of cells. Only a few targeted drugs, such as imatinib and dasatinib, are recommended, based on small-sample studies 15, 16. Despite aggressive treatment, the 10-year overall survival rate for skull base lesions is approximately 60%, and significantly lower for sacral tumors 17. These factors underscore the urgent need for more effective systemic therapies.

The lack of effective curative options for chordoma has necessitated the exploration of alternative strategies. Immune checkpoint inhibitors (ICIs), which have revolutionized treatment across multiple tumor histologies, offer a potential new approach. Antibodies targeting CTLA-4, PD-1 and PD-L1 have been approved for the treatment of melanoma, lung cancer, renal cell carcinoma, and several other malignancies 18-21. Pre-clinical data indicate a complex interaction between chordomas and the immune system, particularly involving PD-1/PD-L1 interactions that may promote chordoma's locally aggressive behavior and immune evasion 22-24. An analysis of 78 chordoma tumor samples revealed a PD-L1 positivity rate of 94.9%, and another study on 58 spinal chordoma samples showed PD-L1 expression in 68.1% of tumors, with a 70.4% expression rate in tumor-infiltrating lymphocytes 22, 23. High PD-L1 expression, linked with poor prognosis in various cases, supports the rationale for testing ICIs in chordoma 25.

In our study, we investigate the molecular characteristics of chordoma using a combination of transcriptomic and computational analyses, including differential expression analysis, gene set enrichment analysis (GSEA), immune cell deconvolution, and weighted gene co-expression network analysis. We hypothesize that chordoma exhibits elevated expression of key immune checkpoints, such as PD-1 and CTLA-4, compared to normal tissue. Additionally, we aim to characterize the immune cell composition within chordoma tumors, with a focus on T cells and macrophages, and identify potential immunogenic subtypes that may respond to immunotherapy. If confirmed, our findings could open up novel avenues for employing ICIs and immunotherapeutic strategies in the treatment of chordoma, an approach that has revolutionized the management of various other types of cancer but remains unexplored in this context 26-30.

## Materials and Methods

### Public data collection and preparation

GSE224776 31, GSE56183 32, and GSE239531 33 were downloaded from the Gene Expression Omnibus (GEO) database. GSE224776 was generated using the Affymetrix Human Genome U133 Plus 2.0 Array, GSE56183 was generated using the Affymetrix Human Gene 1.0 ST Array [probe set (exon) version], and GSE239531 data were generated using the Illumina NovaSeq 6000 platform. For the microarray data, we obtained raw count data, standardized the data using the rma() function in the affy R package 34, annotated the probes in GSE224776 using the hgu133plus2.db R package 35, and annotated the probes in GSE56183 using GPL10739. After merging the two GEO datasets, we performed principal component analysis (PCA) using ggbiplot 36, and corrected batch effects using the removeBatchEffect() function in the limma R package 37. For GSE239531, after acquiring the raw count expression matrix from GEO and merging it with our self-tested RNA-seq dataset, we annotated probes using the biomaRt package based on Homo_sapiens.GRCh38.112 38, 39. Batch correction was performed using Combat-seq in the sva R package 40.

### Sample collection and preparation

Chordoma tissue specimens (n=11) were preserved in RNAlater prior to RNA sequencing at Berry Genomics. Total RNA (1 µg per sample) served as input material for library preparation using NEBNext® Ultra™ RNA Library Prep Kit for Illumina® (NEB, USA) according to manufacturer's protocol. Sequencing was performed on an Illumina NovaSeq platform with paired-end reading strategy. Raw data quality assessment was conducted using FastQC 41, followed by adapter sequence removal using Trim Galore 42. Reads were mapped to the human reference genome using HISAT2, and subsequent quantification was conducted using featureCounts 43, 44.

### Differential expression analysis

Differential expression analysis between chordoma and muscle tissue samples was conducted using the limma R package 37. Genes with adjusted *P*-value < 0.05 and |log2 fold change| > 1 were identified as differentially expressed genes (DEGs). Volcano plots of DEGs were visualized using the EnhancedVolcano R package 45. DEGs with log2 fold change < -1 and adjusted *P*-value < 0.05 were considered downregulated, while those with log2 fold change > 1 and adjusted *P*-value < 0.05 were considered upregulated. The top 5 up- and down-regulated genes are labeled in the figure.

### Gene set enrichment analysis

GSEA was performed using the clusterProfiler R package based on MSigDB collections 46, 47 to investigate potential signaling pathways and biological functions associated with chordoma. Differential transcriptional profiles were identified and sorted by log2 fold change using limma, followed by GSEA enrichment assessment using normalized enrichment score (NES). Results with false discovery rate (FDR) < 0.25 and adjusted P-value < 0.05 were considered statistically significant. The network of interconnected clusters was visualized using the aPEAR package 48, and GSEA enrichment analysis results were visualized using the GseaVis R package 49.

### Immune cell analysis

After merging GSE224776 and GSE56183 datasets, immune and stromal scores were calculated for normal and chordoma samples using the ESTIMATE R package 50. Immune infiltration was assessed using the immunedeconv R package 51 with multiple algorithms: CIBERSORT 52, xCell 53, quanTIseq 54, ABIS 55, EPIC 56, and MCPcounter 57. Immune-related gene sets and immune checkpoint gene sets were obtained from the literature. The ssGSEA algorithm in the GSVA R package was used to score immune genes and immune checkpoints across samples 58. Immunophenotypic scoring was calculated using the IOBR R package 59. Immune infiltration stacked and box plots were generated using the ggplot2 R package, and violin plots were created using the ggpubr R package 60, 61.

### Weighted gene co-expression network analysis

Weighted Gene Co-expression Network Analysis (WGCNA) was used to cluster genes with similar expression patterns and identify gene groups associated with specific traits 62. The coefficient of variation (CV = standard deviation/mean) of 30 samples was used for gene screening. We selected 6,296 genes with CV > 0.1 for WGCNA analysis. Network construction used a soft threshold parameter (β), determined by the lowest power when the scale-free topological model fit reached 0.85. A cluster tree was plotted with different colors representing modules, and highly similar dynamic modules were merged when the tangent was 0.25. Pearson correlation analysis examined relationships between gene modules to identify those most strongly associated with cluster classification. Gene significance (GS) and module membership (MM) values were generated. Genes with GS > 0.8 and MM > 0.4 were selected as key module genes.

### Consensus clustering

Consensus clustering was used to identify molecular subtypes through multiple data subset analysis and comprehensive clustering. We set the maximum number of categories to 6 and performed 1,000 iterations for each *k*. Pearson's coefficient was used as the clustering distance. Cluster number was determined using the ConsensusClusterPlus R package 63. Dimensionality reduction clustering and sample distribution visualization were performed using the Rtsne R package 64, 65.

### Protein-protein interaction

Protein-protein interaction (PPI) networks were constructed using STRING (http://string-db.org) 66. WGCNA-identified candidate genes were entered into STRING, and genes with interaction scores > 0.4 were selected to construct a network model using Cytoscape 67. The MCODE plugin identified core genes, and only interacting genes were selected for further analysis 68.

### Machine learning

Hub gene evaluation employed four distinct machine learning approaches, each selected for their specific strengths in feature selection and ability to manage high-dimensional transcriptomic data: (1) Least Absolute Shrinkage and Selection Operator (LASSO) was chosen for its capacity to perform feature selection through L1 regularization, shrinking coefficients of less important genes to zero, reducing overfitting, and highlighting the most relevant genes; (2) Boruta, a random forest-based method, was selected for its robustness in distinguishing truly predictive genes by comparing them to artificially created shadow features, ensuring only significant contributors are retained; (3) Support Vector Machine - Recursive Feature Elimination (SVM-RFE) was employed due to its effectiveness in ranking genes based on their impact on classification accuracy, iteratively removing less important features to refine the gene set; and (4) eXtreme Gradient Boosting (XGBoost) was included for its superior performance in ranking feature importance via iterative gradient boosting, capturing complex gene interactions and optimizing predictive models. These analyses were implemented using the R packages glmnet 69, caret 70, Boruta 71, and xgboost 72, respectively. The final set of hub genes was determined by intersecting the results from all four algorithms, achieving a consensus on the most critical genes.

### Statistical analysis

All statistical analyses were performed using R software (Version 4.3.1) 31. Spearman's rank correlation test assessed relationships between gene expression and immune cell infiltration. The Wilcoxon test evaluated differences between groups. Results with *P*-value < 0.05 were considered statistically significant.

## Results

### Chordoma microenvironment exhibits marked upregulation of immune checkpoints

**Figure 1:**
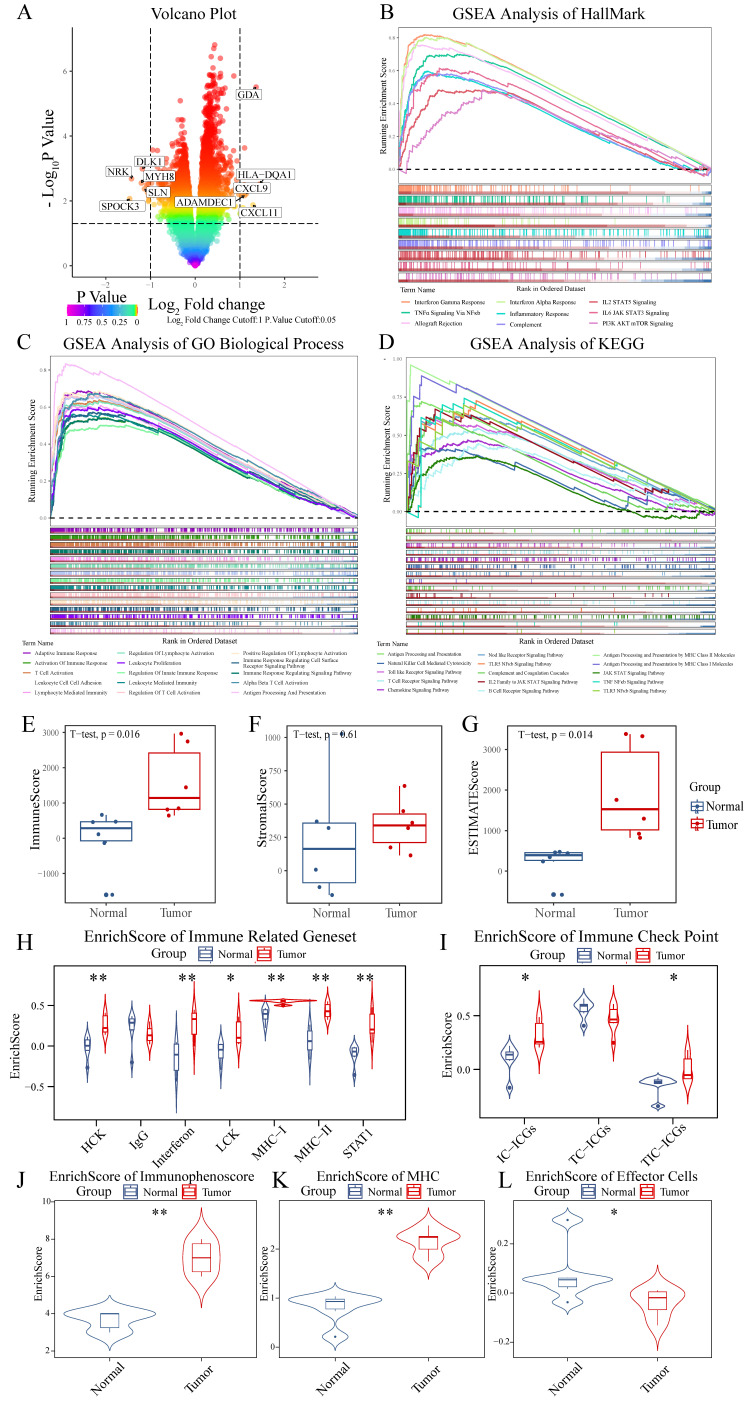
** Transcriptional and immunological profiling reveals enhanced immune signatures in chordoma. (A)** Volcano plot showing differential gene expression between chordoma and normal tissues. Significantly dysregulated genes (|log2 fold change| > 1, P < 0.05) are highlighted, with key upregulated genes (HLA-DQA1, GDA, CXCL11, CXCL9, ADAMDEC1) shown in red and downregulated genes (SPOCK3, NRK, MYH8, DLK1, SLN) in blue. **(B-D)** Gene Set Enrichment Analysis (GSEA) demonstrating enrichment of immune-related pathways in chordoma using different gene set collections: (B) Hallmark gene sets, (C) GO Biological Process, and (D) KEGG pathways. Running enrichment scores (top) and gene set member positions (bottom) are shown for each analysis. **(E-G)** Box plots comparing immune-related scores between normal and tumor tissues (n=6 per group): (E) ImmuneScore, (F) StromalScore, and (G) ESTIMATEScore. Center lines show medians; box limits indicate 25th and 75th percentiles; whiskers extend to 1.5× interquartile range. **(H)** Violin plots showing enrichment scores of seven immune-related gene sets in normal versus tumor samples. **(I)** Distribution of immune checkpoint scores across three categories: TC-ICGs, IC-ICGs, and TIC-ICGs. **(J-L)** Violin plots comparing (J) immunophenoscore, (K) MHC score, and (L) effector cell score between normal and tumor tissues. For all statistical comparisons: *P < 0.05, **P < 0.01, ***P < 0.001.

Analysis of normal tissue and chordoma samples (6 cases each) from GSE224776 and GSE56183 datasets, with batch effect removal using removeBatchEffect from the limma package (Figure S1), revealed distinct differential gene expression patterns as visualized by volcano plots (Figure 1A). The expression analysis identified significant downregulation of SPOCK3, NRK, MYH8, DLK1 and SLN, alongside upregulation of HLA-DQA1, GDA, CXCL11, CXCL9 and ADAMDEC1 in chordomas. To elucidate the biological processes associated with these DEGs, pathway enrichment analysis was performed using GSEA with HallMark, C2 and C5 gene sets from the MSigDB database. These three gene sets provided comprehensive coverage of biological process alterations in chordoma, encompassing tumor hallmarks and common pathway databases including GO, KEGG, Biocarta and Reactome. The analysis revealed enhanced immune-related processes in chordomas compared to normal tissues (Figure 1B-D, Figure S2, Table S1), suggesting crucial involvement of the immune system in chordoma progression through inflammatory responses, antigen presentation and immune cell activation.

The ESTIMATE algorithm was employed for precise assessment of immune system involvement, demonstrating elevated immune scores and Estimate scores in chordoma versus normal tissues (Figure 1E-G, Table S2) 50. Comparison of seven types of immune-related gene sets revealed high enrichment scores for HCK, Interferon, LCK, MHC-I and STAT1 in chordoma (Figure 1H, Table S2) 73. Cancer cells often exploit immune checkpoint mechanisms to escape immune surveillance, with checkpoint expression in the tumor microenvironment classified as tumor cell-dominant (TC-ICGs), immune cell-dominant (IC-ICGs) and tumor and immune cell balanced (TIC-ICGs) 74. Comparative analysis of immune checkpoint scores revealed elevated IC-ICG and TC-ICG scores in the chordoma microenvironment relative to normal tissue (Figure 1I, Table S2). Immunophenotype analysis 75 revealed elevated immune and MHC scores in chordoma tissue, although effector cell scores, which include activated and memory effector CD4 and CD8 cells, were paradoxically decreased (Figure 1J-L, Table S2).

### Chordoma displays selective enrichment of memory T cells and macrophage populations

**Figure 2:**
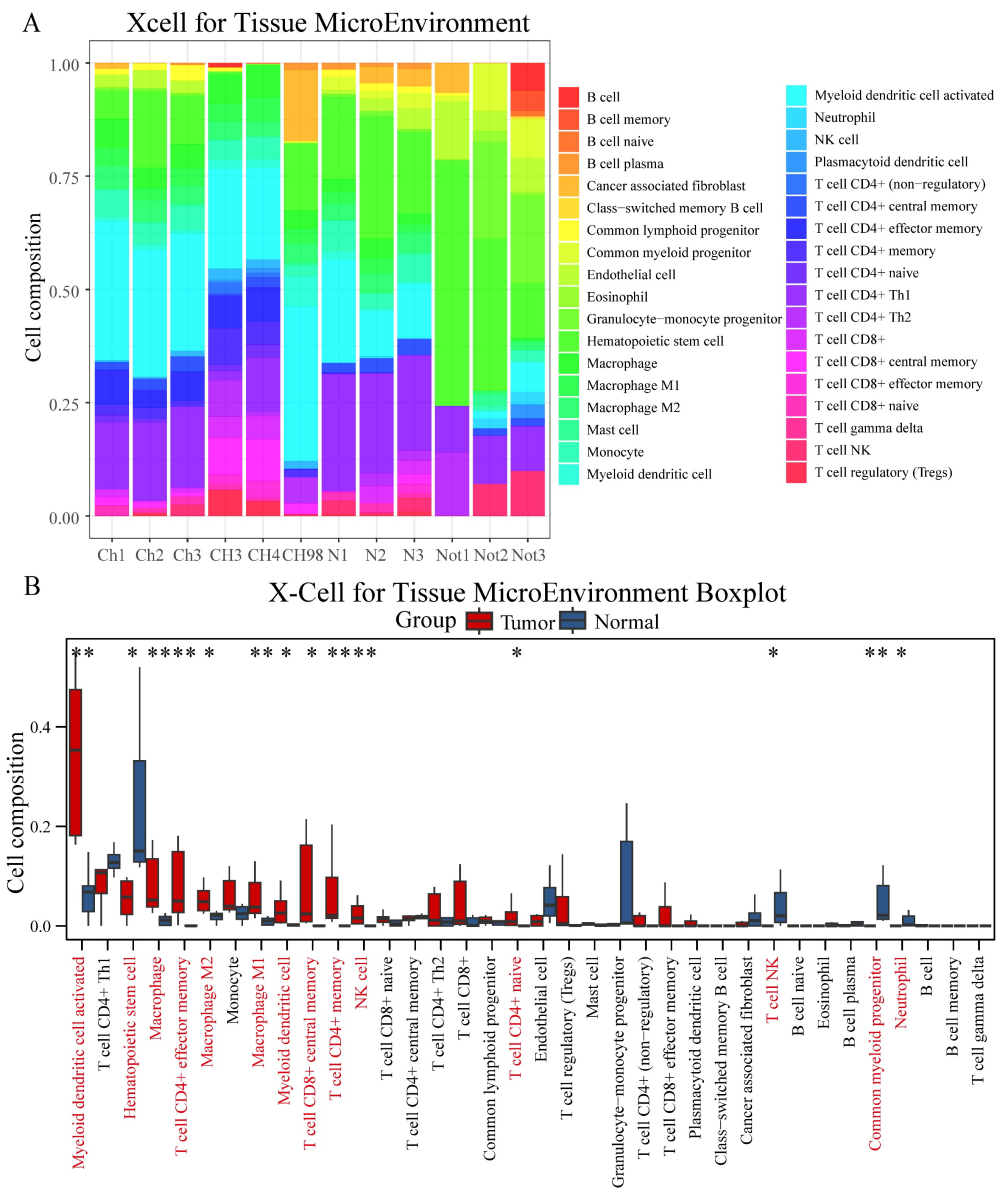
** Comprehensive immune cell profiling reveals distinct infiltration patterns in chordoma. (A)** Stacked bar plot showing the relative proportions of immune cell populations in chordoma (Ch1-CH98) and normal (N1-Not3) samples as determined by XCell algorithm. Colors represent different immune cell types. **(B)** Box plot comparison of immune cell type abundances between normal and tumor tissues. Red bars indicate tumor samples; blue bars indicate normal samples. Significant differences are marked with asterisks (*P < 0.05, **P < 0.01, ***P < 0.001, ****P < 0.0001). Box plot elements as in Figure 1.

Comprehensive immune cell profiling using six distinct algorithms revealed specific patterns of immune cell infiltration in chordoma. XCell analysis demonstrated significantly higher infiltration of multiple immune cell populations in chordoma compared to normal tissue, particularly myeloid dendritic cells, macrophages (M1 and M2), NK cells, and various T cell subsets (CD8+ central memory, CD4+ memory, CD4+ effector memory, and CD4+ naive) (Figure 2A-B, Table S2). These findings were corroborated by multiple alternative algorithms: Cibersort confirmed increased M1 and M2 macrophage infiltration; Abis showed elevated monocyte and memory T cell populations; MCP Counter indicated enhanced monocyte lineages; and Epic demonstrated increased CD4+ T cell and macrophage presence (Figure S3, Table S2).

### Distinct immune cell clustering reveals a highly immunogenic chordoma subtype

**Figure 3:**
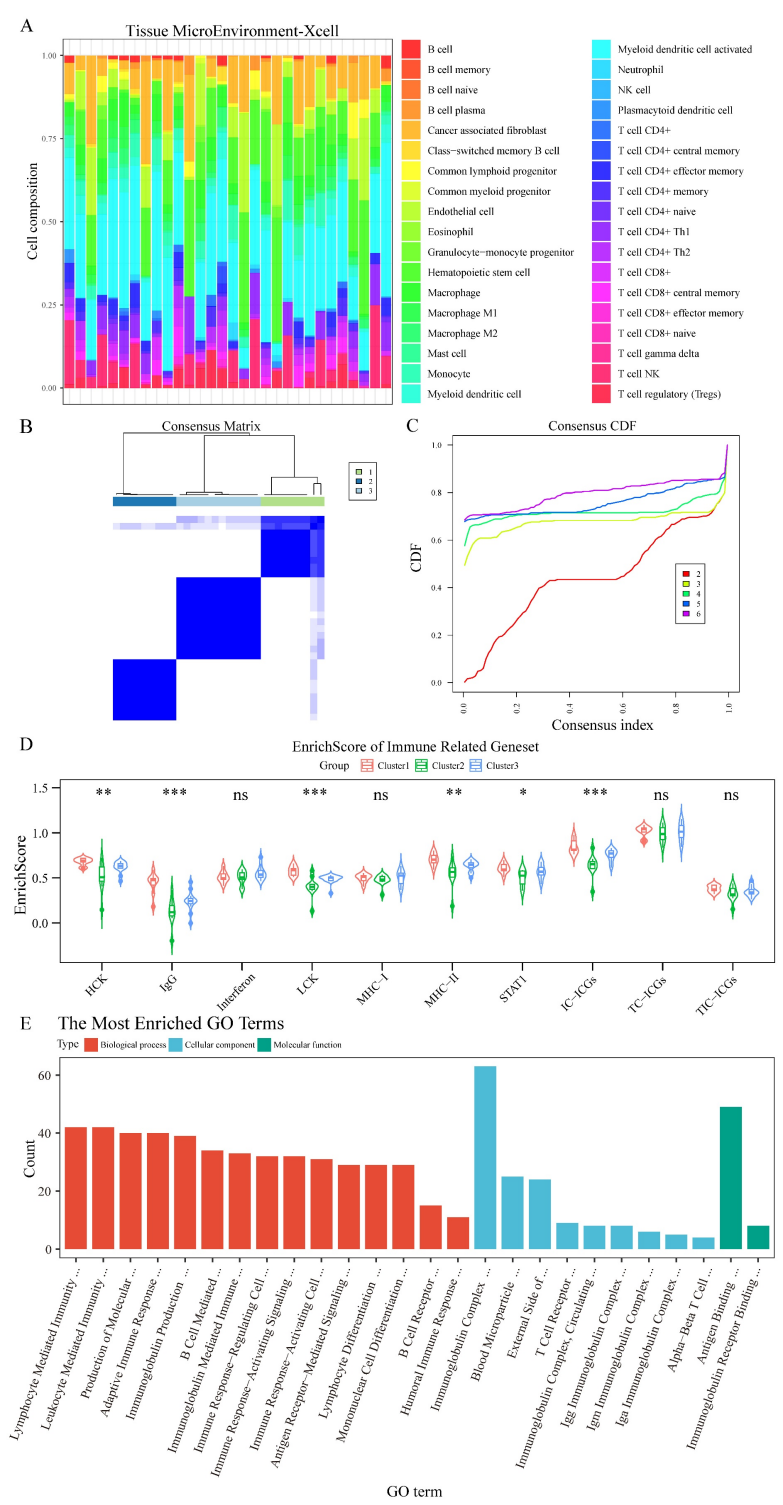
** Consensus clustering identifies a distinct immune-enriched chordoma subtype. (A)** Heatmap showing immune cell composition across 32 chordoma samples based on XCell analysis. **(B)** Consensus matrix for k=3 clustering shows robust cluster separation. **(C)** Cumulative distribution function (CDF) curves validating optimal cluster number selection. **(D)** Violin plots comparing immune-related gene set enrichment scores across three identified clusters. Cluster1 shows distinctly higher scores for multiple immune signatures. Statistical significance indicated as in Figure 1. **(E)** Bar plot showing the most enriched GO terms in Cluster1, categorized by biological process (red), cellular component (blue), and molecular function (green). Length of bars indicates gene count for each term.

Integration of RNA sequencing data from 12 chordoma patients with the GSE239531 dataset (20 chordoma tissues) enabled comprehensive immune cell profiling. Following outlier removal through tree-based clustering and batch effect correction (Figure S4A-D), XCell-based immune cell profiling and consensus cluster analysis (Figure 3A-C) identified three distinct clusters, clearly differentiated in tSNE visualization (Figure S4E).

Cluster1 demonstrated significantly higher scores for immune-related genes (HCK, IgG, LCK, MHC-II, STAT1) and enhanced immune checkpoint expression by immune cells (Figure 3D, Table S3). Differential gene analysis (Figure S4F) followed by GO enrichment analysis revealed Cluster1's enhanced involvement in immune system regulation (Figure 3E, Table S4), particularly in lymphocyte-mediated immunity, antigen receptor signaling, and immune response pathways. GSEA further confirmed Cluster1's enrichment in lymphocyte-mediated immunity regulation and immunoglobulin-mediated immune response (Figure S5A, Table S4), with significant involvement in TCR and IL-12 signaling pathways demonstrated through KEGG, Biocarta, and PID datasets (Figure S5B-D, Table S4).

### WGCNA identifies discrete gene modules governing immune response in chordoma

**Figure 4:**
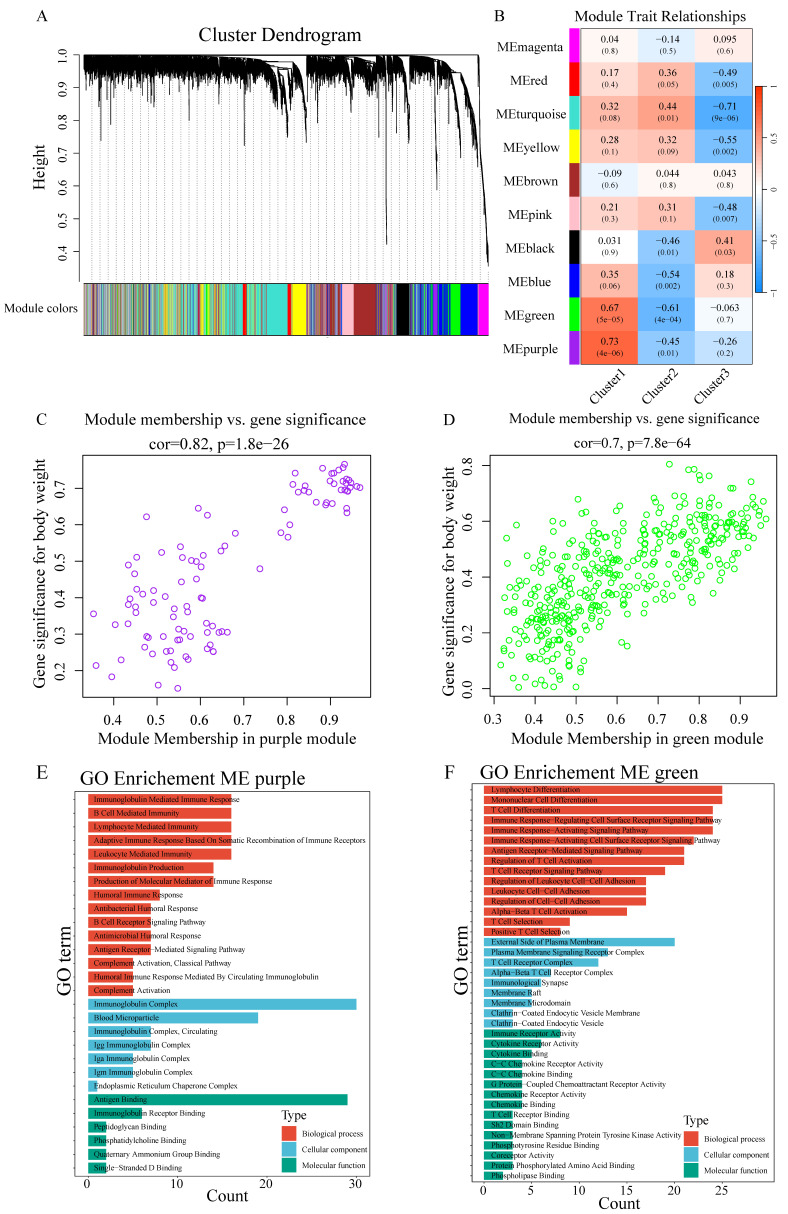
** WGCNA identifies immune-related gene modules in chordoma. (A)** Cluster dendrogram showing gene modules identified by WGCNA. Different colors represent distinct modules. **(B)** Heatmap showing module-trait relationships. Colors indicate correlation strength (red = positive, blue = negative); numbers in parentheses show P-values. **(C,D)** Scatter plots showing correlation between module membership and gene significance for (C) MEpurple module (cor=0.82, P=1.8e-26) and (D) MEgreen module (cor=0.7, P=7.8e-64). **(E,F)** GO enrichment analysis of significant genes in (E) MEpurple and (F) MEgreen modules, categorized by biological process (red), cellular component (blue), and molecular function (green).

WGCNA-based scale-free network construction was performed to identify immune infiltration-associated gene modules using immune cell cluster classification (Figure S6A). To ensure the network adhered to scale-free topology, a soft-thresholding power of β=7 was selected, achieving R² > 0.85 (Figure S6B). The analysis workflow involved transforming the gene expression matrix into an adjacency matrix, followed by topological matrix conversion. Gene modules were identified using hierarchical clustering based on average connectivity, and dynamic tree cutting was applied with a minimum module size threshold of 50 genes. Dynamic pruning methodology identified 10 distinct modules through characteristic gene value calculation (Figure 4A), with MEpurple (R=0.73, p=4e-06) and MEgreen (R=0.67, p=5e-05) showing strong Cluster1 correlation (Figure 4B). Gene significance (GS>0.6) and module membership (MM>0.8) analysis identified key Cluster1-associated genes (Figure 4C-D). GO enrichment analysis revealed MEpurple module's association with B cell function, particularly immunoglobulin-mediated immune response and B cell receptor signaling, while MEgreen module demonstrated involvement in T cell-related processes, including differentiation and receptor signaling (Figure 4E-F).

### Integrated network analysis and machine learning converge on CCR7 as a central immune regulator

**Figure 5:**
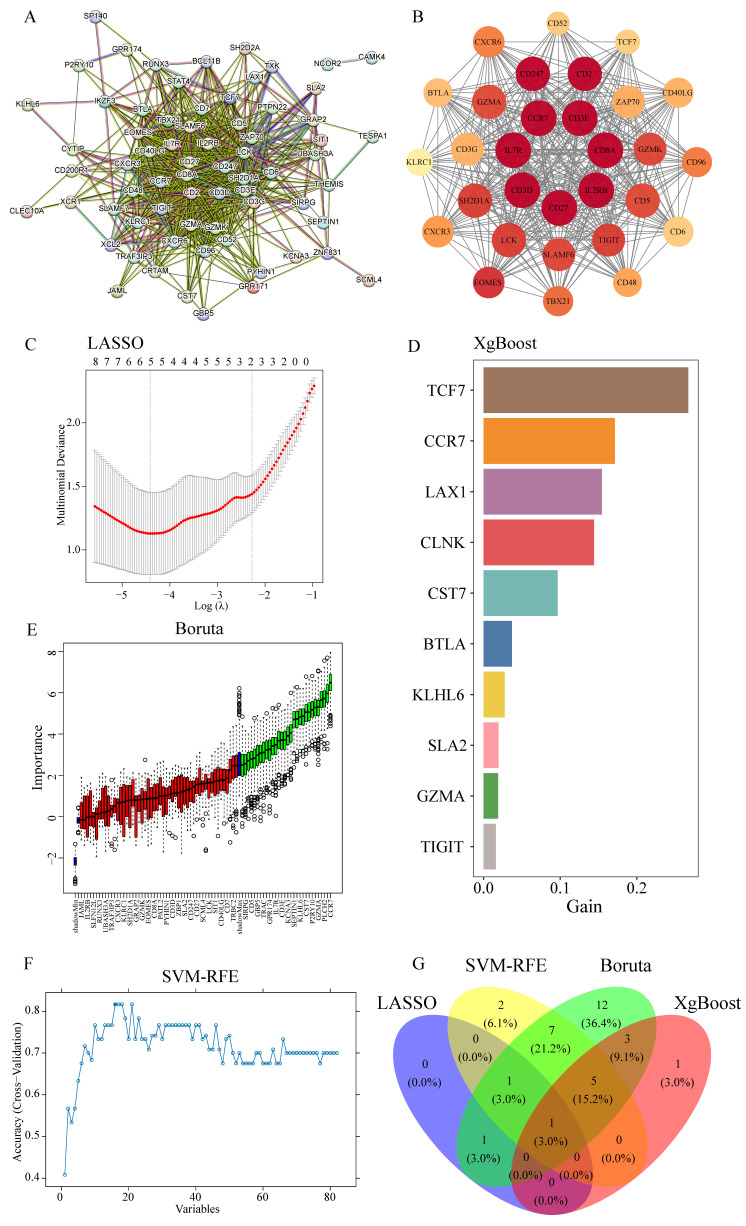
** Integration of PPI network analysis and machine learning identifies CCR7 as a key immune regulator. (A)** PPI network of MEgreen module genes. Node size reflects connectivity degree. **(B)** High-scoring cluster identified by MCODE algorithm showing core immune-related genes. Node color intensity indicates degree of connectivity. **(C)** LASSO regression analysis showing optimal parameter selection. **(D)** Top 10 genes identified by XGBoost algorithm, ranked by importance score (gain). **(E)** Boruta algorithm results showing confirmed (green), tentative (red), and rejected (black) features. **(F)** SVM-RFE performance curve showing classification accuracy across different numbers of features. **(G)** Venn diagram showing overlap of genes identified by four machine learning approaches, highlighting CCR7 as the common candidate.

Given the crucial role of T cells in tumor-directed cytotoxicity 76, PPI network analysis of MEgreen module genes using the MCODE algorithm in Cytoscape identified core network components (Figure 5A). The highest-scoring cluster (score: 27,448) yielded seven key genes through connectivity degree analysis: IL2RB, CD8A, CD3E, IL7R, CD27, CD3D and CCR7 (Figure 5B). Parallel analysis employing four machine learning algorithms provided complementary candidate gene identification: LASSO algorithm identified three candidates (Figure 5C); XGBoost analysis yielded 30 candidates, with the top 10 designated as key genes (Figure 5D); Boruta algorithm identified 30 candidates (Figure 5E); and SVM-RFE analysis produced 16 candidate genes (Figure 5F). Integration of results across all four algorithms identified CCR7 as the sole common candidate gene (Figure 5G).

### Core T cell-associated genes correlate with immune cell distribution patterns

**Figure 6:**
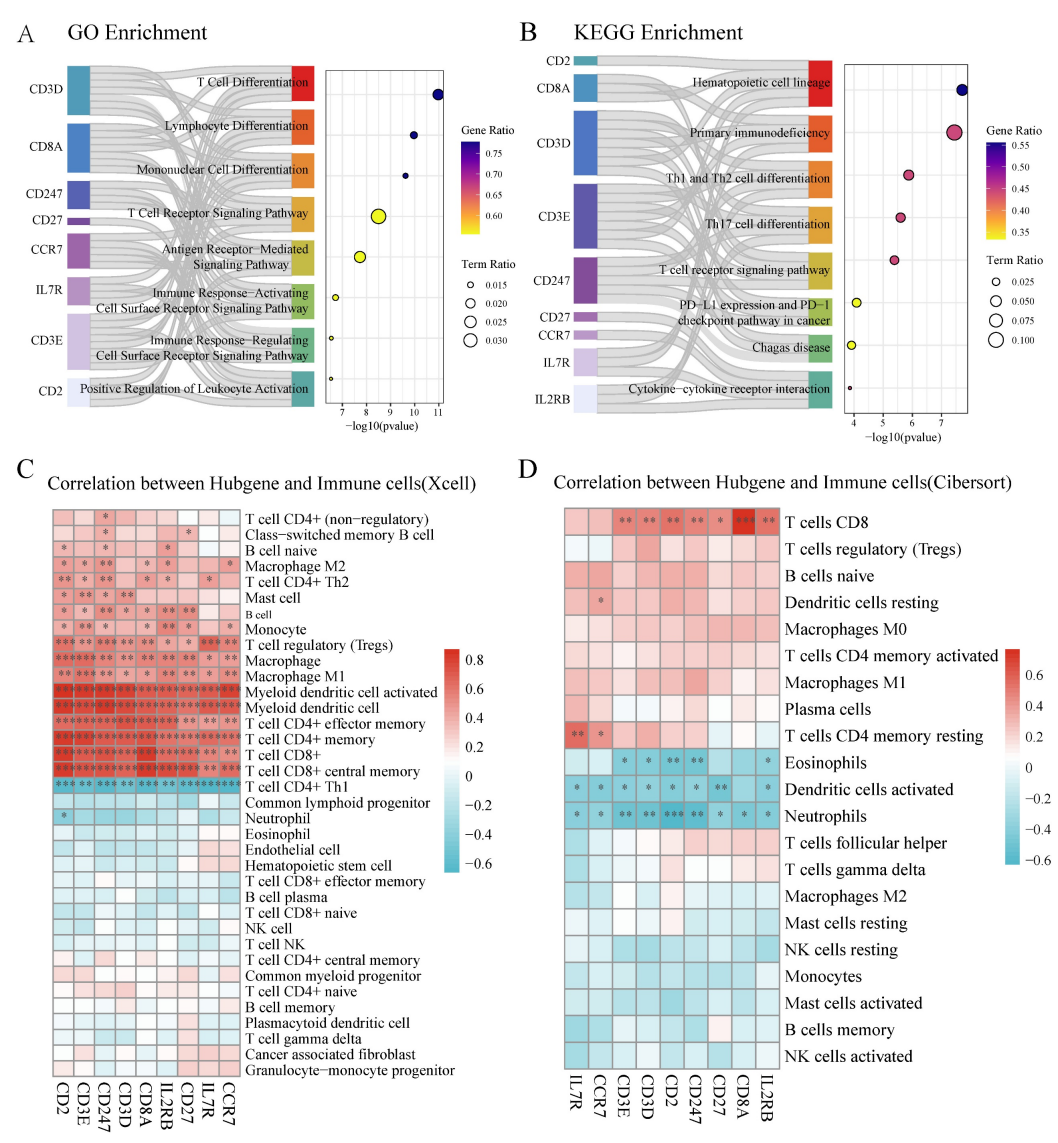
** Core T cell-associated genes demonstrate significant correlation with immune cell populations. (A)** GO enrichment analysis of core genes showing significant pathways related to T cell function. Left bars indicate gene sets, with bar lengths representing gene ratios. Circle size indicates term ratio, and color gradient shows gene ratio. **(B)** KEGG pathway enrichment analysis highlighting involvement in hematopoietic and immune-related pathways. Circle size and color coding follow the same scheme as in (A). **(C)** Correlation heatmap between hub genes and immune cell populations based on XCell algorithm. Color intensity indicates correlation strength (red = positive, blue = negative); asterisks denote statistical significance (*P < 0.05, **P < 0.01). **(D)** Correlation heatmap between hub genes and immune cell populations based on Cibersort algorithm, showing distinct correlation patterns particularly with T cell subsets.

Functional analysis of the identified core genes revealed significant enrichment in T cell differentiation, lymphocyte differentiation, and T cell receptor signaling pathways (Figure 6A, Table S5). KEGG pathway analysis highlighted their involvement in hematopoietic cell lineage, primary immunodeficiency, and PD-L1/PD-1 checkpoint pathways (Figure 6B, Table S5). Correlation analysis demonstrated strong positive associations between most core genes and CD4+/CD8+ T cell populations in XCell analysis (Figure 6C), while Cibersort analysis showed positive correlations between most core genes (except CCR7 and IL7R) and CD8+ T cell distribution (Figure 6D).

## Discussion

The present study provides novel insights into the immune landscape of chordoma, a rare and challenging malignancy. Our findings reveal a complex interplay of immune checkpoints and immune cell populations within the chordoma microenvironment. Most notably, we observed elevated expression levels of key immune checkpoints, including PD-1 and CTLA-4, suggesting the potential for immunotherapeutic interventions targeting these pathways.

The upregulation of immune checkpoints in chordoma is consistent with the immune evasion strategies employed by many cancers. These checkpoints serve as "brakes" on the immune system, preventing excessive immune activation and autoimmunity under normal conditions. However, in the context of cancer, they can be co-opted to suppress anti-tumor immune responses. The high expression of PD-1 and CTLA-4 in our chordoma samples suggests that these tumors may be particularly adept at exploiting these pathways to evade immune surveillance.

Despite the elevated immune checkpoint levels, the efficacy of ICIs in chordoma treatment remains uncertain. Several factors may influence the response to ICIs, including tumor mutational burden (TMB), PD-L1 expression, and the composition of the immune microenvironment. Chordomas typically exhibit a low TMB, which has been associated with reduced responsiveness to ICIs in other cancers 77, 78. However, recent evidence suggests that even low-TMB tumors, such as pancreatic and ovarian cancers, can respond to ICIs under certain conditions 79, 80. Therefore, the low TMB of chordomas does not necessarily preclude the use of ICIs in their treatment.

Recent clinical trials exploring immunotherapy in chordoma have focused on ICIs like nivolumab and pembrolizumab, especially after vaccine therapies targeting brachyury were proved ineffective 81, 82. Advances in targeting the PD-1/PD-L1 pathway have led to significant and sustained clinical improvements in several types of advanced human cancers. PD-1/PD-L1 ICIs are also the most commonly used drugs in chordoma patients 83-85.

In addition to the immune checkpoint landscape, our study also sheds light on the immune cell composition within chordoma tumors. We observed a downregulation of neutrophils and an increased presence of resting NK cells compared to normal muscle tissue. Neutrophils play a complex role in the tumor microenvironment, with both pro- and anti-tumor effects depending on the context. The downregulation of neutrophils in chordoma may reflect an immunosuppressive microenvironment that limits their recruitment and activation. Conversely, the increased presence of resting NK cells suggests a potential reservoir of cytotoxic effector cells that could be harnessed for anti-tumor immunity. However, the functional status of these NK cells and their ability to mount an effective response against chordoma cells remain to be determined.

The identification of distinct immune cell clusters within chordoma tumors further highlights the heterogeneity of the immune microenvironment. Cluster 1, characterized by elevated expression of immune-related genes and immune checkpoints, represents a highly immunogenic subtype of chordoma. This finding aligns with the concept of "hot" tumors, which are more likely to respond to immunotherapies due to their pre-existing immune infiltration and activation. The enrichment of lymphocyte-mediated immunity and immunoglobulin-mediated immune response pathways in Cluster 1 suggests a potential role for B cell-mediated anti-tumor immunity in this subtype.

The application of weighted gene co-expression network analysis (WGCNA) and machine learning approaches in our study allowed for the identification of key immune-related gene modules and hub genes associated with the immunogenic Cluster 1. The MEpurple module, enriched in B cell receptor signaling and immunoglobulin-mediated immune response, and the MEgreen module, involved in T cell differentiation and receptor signaling, provide potential targets for future investigations into the molecular mechanisms underlying the immune response in chordoma. The convergence of network analysis and machine learning on CCR7 as a central immune regulator in chordoma is particularly intriguing. CCR7 is a chemokine receptor involved in lymphocyte migration and homing to secondary lymphoid organs. Its upregulation in chordoma may reflect an active immune response and the recruitment of immune cells to the tumor site. The correlation of CCR7 and other core T cell-associated genes with CD4+ and CD8+ T cell infiltration further supports their functional relevance in the chordoma immune microenvironment.

Our study faced several limitations inherent to the study of rare cancers like chordoma. The limited availability of tissue samples necessitated the use of adjacent muscle tissue as a proxy for normal controls, which may not perfectly mirror the properties of peritumoral tissue. Additionally, the small sample size, while statistically justified, may not fully capture the heterogeneity within chordomas. Despite these limitations, our findings provide valuable insights into the immune landscape of chordoma and lay the groundwork for future investigations.

Moving forward, several avenues of research could build upon our findings. First, the functional significance of the elevated immune checkpoints and the immunogenic Cluster 1 subtype should be investigated using *in vitro* and *in vivo* models. Blocking these checkpoints with ICIs and assessing the impact on tumor growth and immune cell activation could provide direct evidence of their role in chordoma immune evasion. Second, the mechanisms underlying the recruitment and activation of macrophages and memory T cells in chordoma warrant further exploration. Understanding the factors that shape the immune cell composition within these tumors could inform strategies to modulate the microenvironment and enhance anti-tumor immunity.

Third, clinical trials evaluating the safety and efficacy of ICIs in chordoma patients are needed to translate our findings into potential therapeutic applications. While recent trials have explored ICIs like nivolumab and pembrolizumab in chordoma, the factors influencing response remain unclear. Bishop *et al.* noted that 88% of chordoma patients treated with ICIs experienced clinical benefits, including about a quarter who achieved partial or complete responses. However, the factors behind these improved outcomes have remained unclear - whether it's the type of ICI used, expression of tumor checkpoints, genetic characteristics of the tumor, the immune microenvironment, or other patient- and tumor-specific variables. Future trials should incorporate comprehensive immune profiling, including assessment of immune checkpoint expression, TMB, and immune cell infiltration, to identify predictors of response and guide patient selection. Additionally, combination therapies targeting multiple immune checkpoints or combining ICIs with other treatment modalities, such as radiation or targeted therapies, should be explored in chordoma. The complex immunosuppressive strategies employed by these tumors may require a multi-faceted approach to effectively overcome immune evasion and promote anti-tumor immunity.

The application of ICIs in chordoma treatment presents with a unique set of challenges. Due to the rarity of chordomas, they often are not prioritized in drug development 12. Furthermore, the effectiveness of ICIs often varies among patients, necessitating further research to identify the factors influencing response in this context 4, 86. Bishop *et al.* noted that 88% of chordoma patients treated with ICIs experienced clinical benefits, including about a quarter who achieved partial or complete responses 86. However, the factors behind these improved outcomes have remained unclear - whether it's the type of ICI used, expression of tumor checkpoints, genetic characteristics of the tumor, the immune microenvironment, or other patient- and tumor-specific variables.

Historically, chordoma research has been centered on tumor cell mechanisms, with limited insights into the tumor's immune microenvironment. Only recently, studies have highlighted the prominence of macrophages and T cells in chordoma. Our research contributes to this evolving understanding, including by demonstrating neutrophil downregulation and increased presence of resting NK cells within chordoma tumors. In tumors, neutrophils attack and modulate the microenvironment, while resting NK cells surveil and eliminate tumor cells through cytotoxic responses. Yet, their effectiveness is likely compromised by chordoma's complex immunosuppressive strategies. However, the more specific mechanisms of immune evasion and suppression in chordomas are not well defined. Thus, exploring the molecular mechanisms of the tumor microenvironment and assessing the effectiveness of dual-target immunotherapy are critical next steps in chordoma research. This approach may offer improved outcomes over treatments focusing solely on ICIs.

## Conclusion

This study reveals a complex immune microenvironment in chordoma, characterized by elevated immune checkpoint expression (e.g., PD-1, CTLA-4), a highly immunogenic subtype (Cluster 1), and a T cell-centric regulatory network with CCR7 as a key modulator. These findings highlight the potential of immune checkpoint inhibitors (ICIs) and T cell-targeted therapies as viable strategies for chordoma treatment. By identifying actionable immune signatures, this work paves the way for personalized immunotherapeutic approaches, offering hope for improved outcomes in this challenging malignancy where conventional therapies fall short. Future efforts should focus on validating these targets in clinical trials to translate these insights into effective treatments.

## Supplementary Material

Supplementary figures and tables.
