# Supplementary material for: Cracking Chordoma's Conundrum: Immune Checkpoints Provide a Potential Modality
Source: Int J Med Sci. 2025 Apr 22;22(10):2318–32. doi: 10.7150/ijms.109721 (PMC12080586; doi:10.7150/ijms.109721)
Supplement: Supplementary file 1 — Supplementary figures and tables. [file ijmsv22p2318s1.zip › Supplementary materials.pdf]

Figure S1 | Batch effect removal and data integration of microarray datasets. (A) rincipal Component Analysis (PCA) plot before batch effect removal showing clear separation between normal and tumor samples, with PC1 explaining 85.8% and PC2 explaining 5.8% of variation. **(B)** Box plots showing expression distribution across samples from different datasets (GSE224776 and GSE56183) before batch correction. **(C)** PCA plot after batch effect removal demonstrating successful integration of datasets, with PC1 explaining 41.1% and PC2 explaining 20.6% of variation. **(D)** Box plots showing normalized expression distribution after batch effect removal.

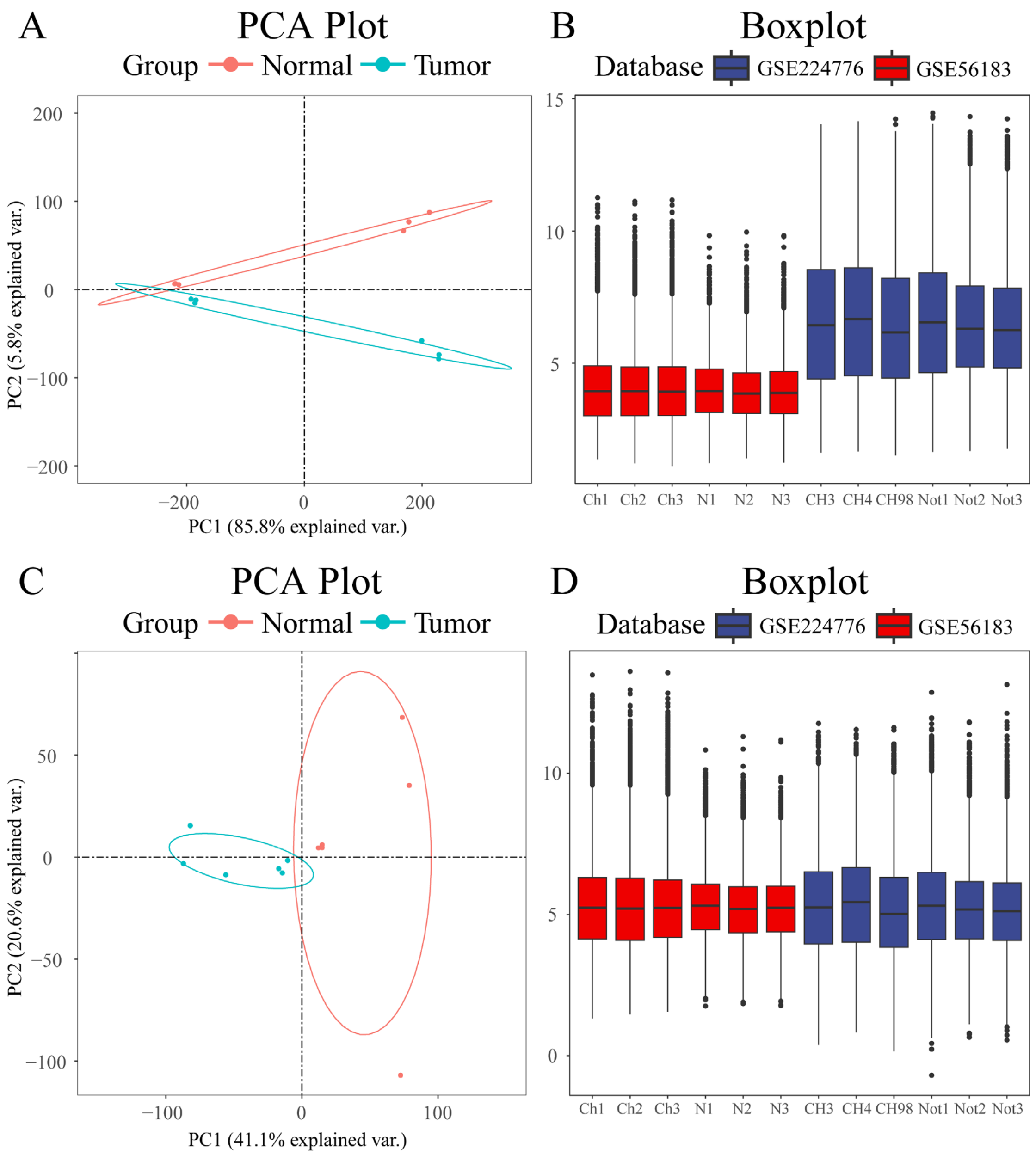
A
PCA Plot
Group Normal Tumor
PC2 (5.8% explained var.)
PC1 (85.8% explained var.)
200
100
0
−100
−200
−200
0
200
B
Boxplot
Database GSE224776 GSE56183
15
10
5
Ch1 Ch2 Ch3 N1 N2 N3 CH3 CH4 CH98 Not1 Not2 Not3
C
PCA Plot
Group Normal Tumor
PC2 (20.6% explained var.)
PC1 (41.1% explained var.)
50
0
−50
−100
−100
0
100
D
Boxplot
Database GSE224776 GSE56183
10
5
0
Ch1 Ch2 Ch3 N1 N2 N3 CH3 CH4 CH98 Not1 Not2 Not3

Figure S2 | Extended pathway analysis reveals comprehensive immune-related signatures. (A) GSEA analysis using BIOCARTA database showing enrichment of T cell-related pathways including T cytotoxic, T helper, CTL, and TCR signaling pathways. **(B)** GSEA analysis using PID database demonstrating enrichment of IL-12, CD8 TCR, IL-2, and NFκB canonical pathways. **(C)** GSEA analysis using REACTOME database highlighting enrichment of interferon and antigen processing pathways. **(D)** GSEA analysis using WikiPathways showing enrichment of T cell receptor signaling and cytokine-related pathways.

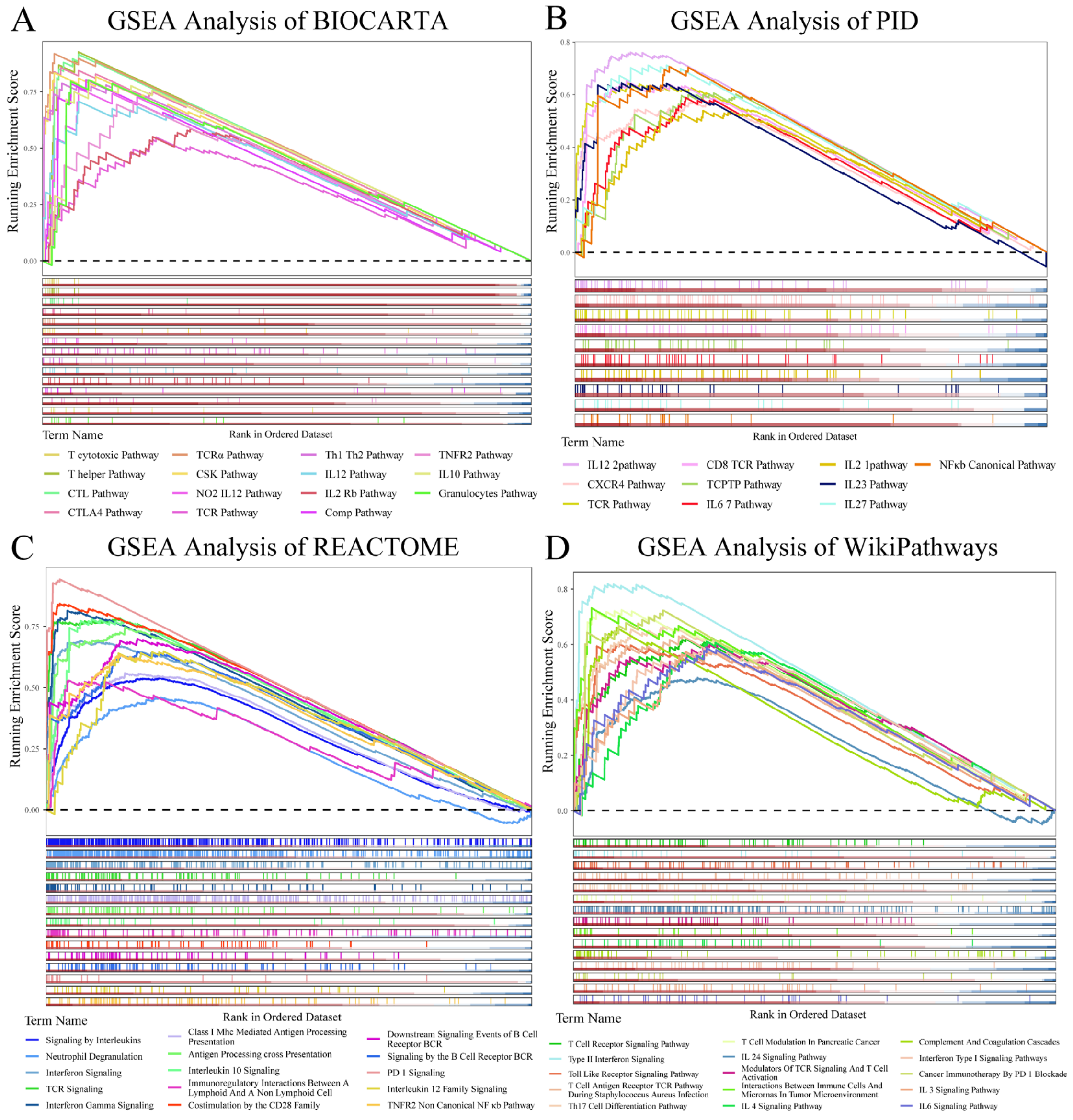

Figure S3 | Multi-algorithm validation of immune cell infiltration patterns.

Comprehensive immune cell composition analysis using five different computational approaches. From top to bottom: Cibersort showing distinct patterns of immune cell infiltration between tumor and normal samples; Quantiseq demonstrating differential immune cell populations; Abis revealing significant differences in T cell and macrophage populations; MCP Counter highlighting monocyte lineage differences; and Epic showing distinct T cell and macrophage patterns. Red boxes indicate tumor samples, blue boxes indicate normal samples. Statistical significance: *$P < 0.05$, **$P < 0.01$.

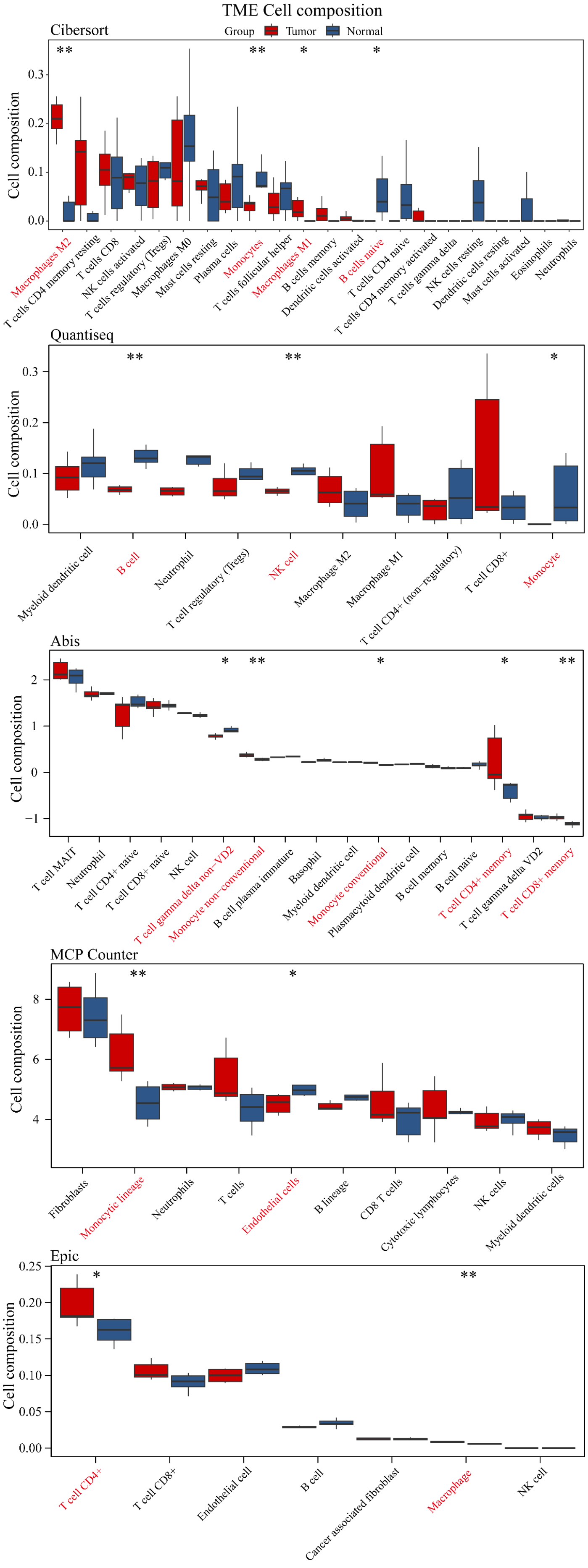

TME Cell composition
Group
Tumor
Normal
Cibersort
Cell composition
Macrophages M2
T cells CD4 memory resting
T cells CD8
NK cells activated
T cells regulatory (Tregs)
Macrophages M0
Mast cells resting
Plasma cells
Monocytes
T cells follicular helper
Macrophages M1
B cells memory
Dendritic cells activated
B cells naive
T cells CD4 naive
T cells CD4 memory activated
T cells gamma delta
NK cells resting
Dendritic cells resting
Mast cells activated
Eosinophils
Neutrophils
Quantiseq
Cell composition
Myeloid dendritic cell
B cell
Neutrophil
T cell regulatory (Tregs)
NK cell
Macrophage M2
Macrophage M1
T cell CD4+ (non−regulatory)
T cell CD8+
Monocyte
Abis
Cell composition
T cell MAIT
Neutrophil
T cell CD4+ naive
T cell CD8+ naive
NK cell
T cell gamma delta non−VD2
Monocyte non−conventional
B cell plasma immature
Basophil
Myeloid dendritic cell
Monocyte conventional
Plasmacytoid dendritic cell
B cell memory
B cell naive
T cell CD4+ memory
T cell gamma delta VD2
T cell CD8+ memory
MCP Counter
Cell composition
Fibroblasts
Monocytic lineage
Neutrophils
T cells
Endothelial cells
B lineage
CD8 T cells
Cytotoxic lymphocytes
NK cells
Myeloid dendritic cells
Epic
Cell composition
T cell CD4+
T cell CD8+
Endothelial cell
B cell
Cancer associated fibroblast
Macrophage
NK cell

Figure S4 | Sample clustering and differential expression analysis. (A,B) Hierarchical clustering dendrograms showing sample relationships before (A) and after (B) outlier removal. **(C,D)** Principal component analysis plots showing sample distribution from different sources (GEO and PKU) before (C) and after (D) batch effect correction. **(E)** tSNE plot showing three distinct clusters identified through consensus clustering. **(F)** Volcano plot of differential expression analysis between clusters, with significantly altered genes highlighted ($|\log2 \text{ fold change}| > 1$, adjusted $P < 0.05$).

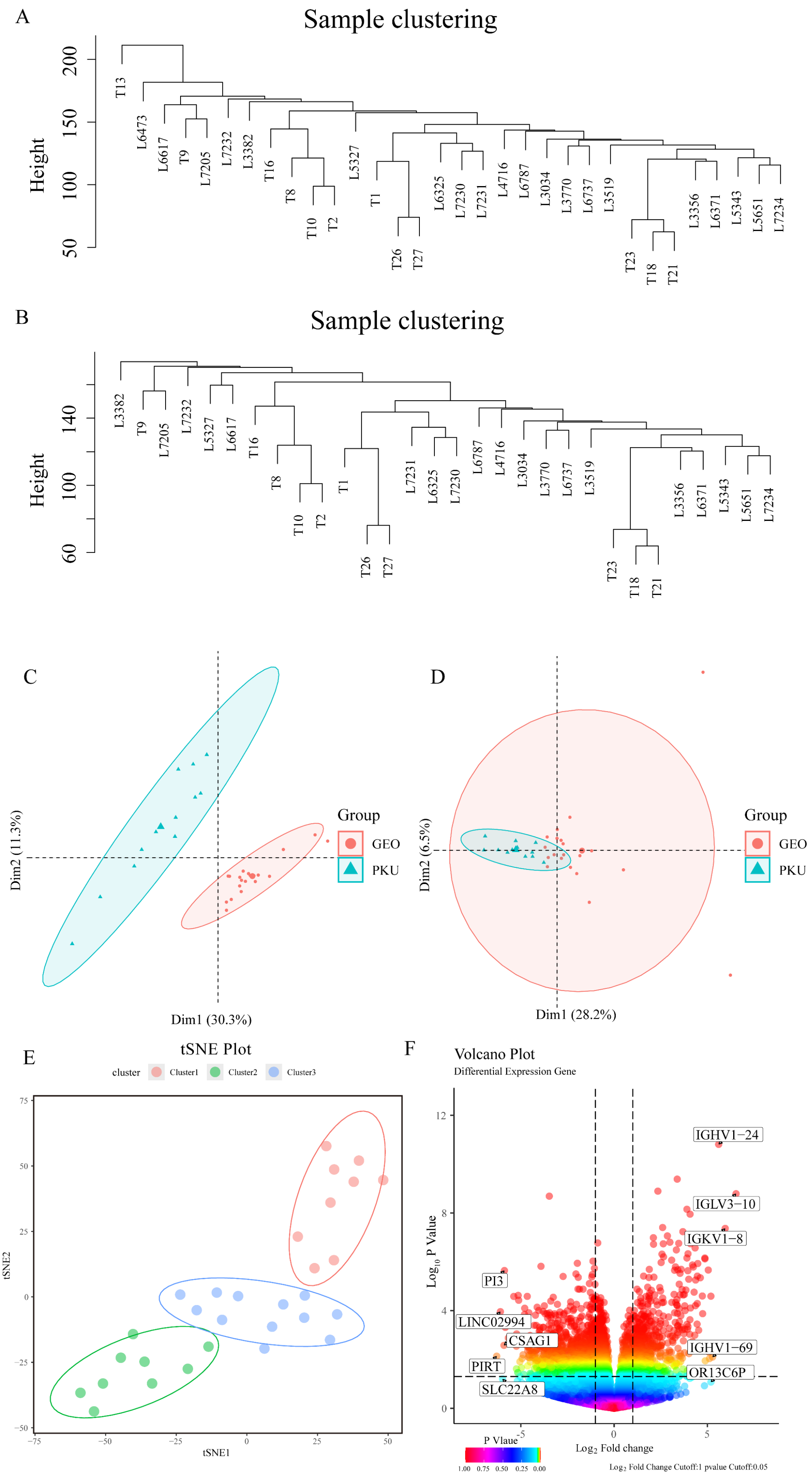
A
Sample clustering
Height
B
Sample clustering
Height
C
Dim1 (30.3%)
Dim2 (11.3%)
Group
GEO
PKU
D
Dim1 (28.2%)
Dim2 (6.5%)
Group
GEO
PKU
E
tSNE Plot
cluster Cluster1 Cluster2 Cluster3
tSNE1
tSNE2
F
Volcano Plot
Differential Expression Gene
IGHV1−24
IGLV3−10
IGKV1−8
PI3
LINC02994
CSAG1
PIRT
SLC22A8
IGHV1−69
OR13C6P
Log2 Fold change
P Vlaue
Log2 Fold Change Cutoff:1 pvalue Cutoff:0.05

Figure S5 | Network and enrichment analysis demonstrates interconnected immune pathways in chordoma. (A) Network visualization of enriched GO Biological Process terms. Node size represents pathway size, color intensity indicates normalized enrichment score (NES), and connecting lines show relationships between pathways. The network reveals major clusters centered around lymphocyte-mediated immunity, T cell activation, and leukocyte differentiation, with oxidative phosphorylation showing distinct separation. **(B)** KEGG pathway network analysis displaying interconnected immune signaling pathways. The TCR pathway forms a central hub connected to IL-12, NFAT TF, BCR, Th1/Th2, and IL-2 STAT5 pathways. Node size corresponds to pathway size, and color intensity indicates NES values. **(C)** GSEA analysis using BIOCARTA database showing running enrichment scores for multiple immune-related pathways, including T cytotoxic, T helper, TCR, and IL pathways. **(D)** GSEA analysis using PID database demonstrating enrichment patterns of immune signaling pathways, particularly in TCR, IL-12, and NFκB signaling cascades. Both C and D include gene set position plots (bottom) showing the distribution of pathway members across the ranked dataset.

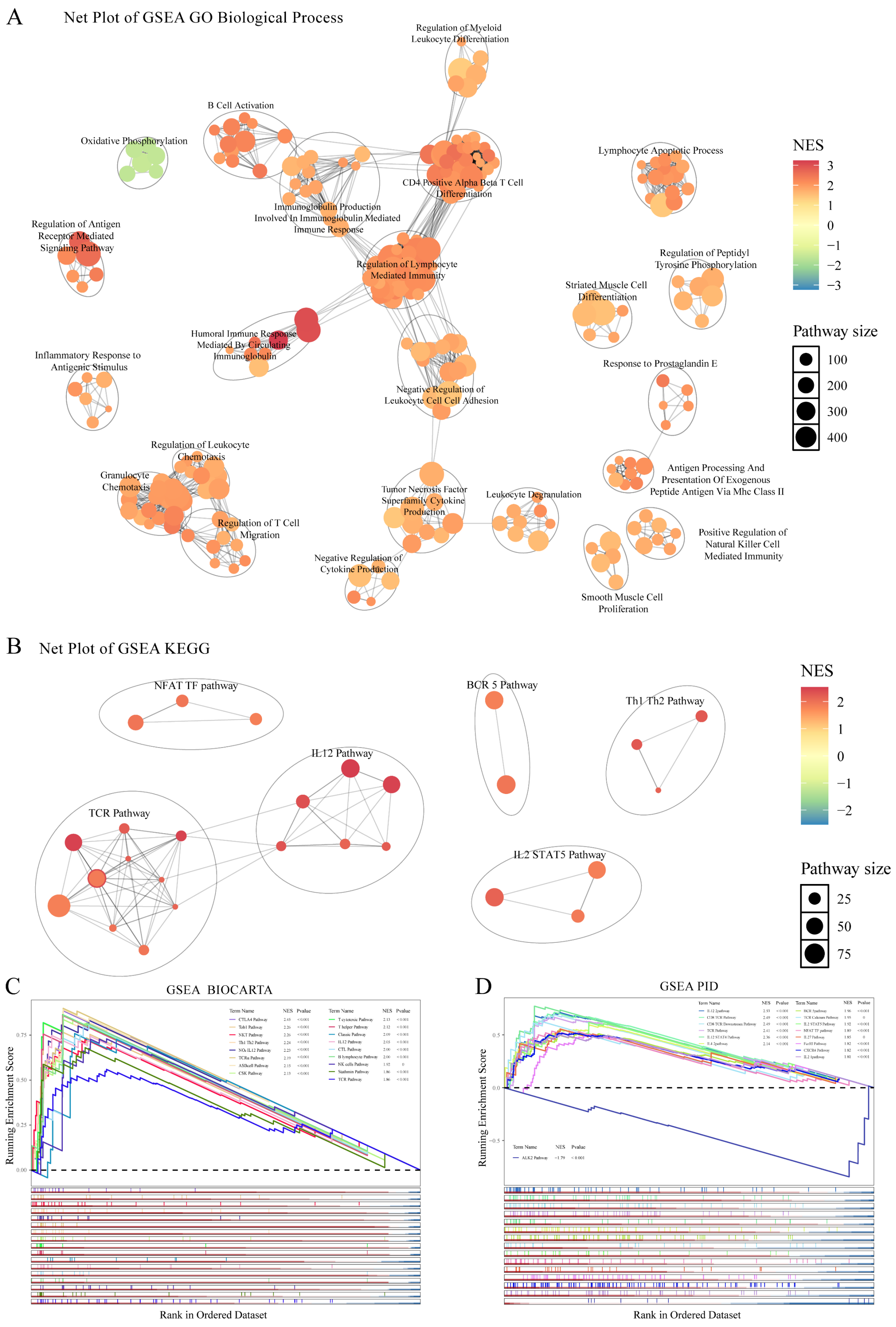
A
Net Plot of GSEA GO Biological Process
Regulation of Myeloid Leukocyte Differentiation
B Cell Activation
Oxidative Phosphorylation
Lymphocyte Apoptotic Process
CD4 Positive Alpha Beta T Cell Differentiation
Immunoglobulin Production Involved In Immunoglobulin Mediated Immune Response
Regulation of Antigen Receptor Mediated Signaling Pathway
Regulation of Lymphocyte Mediated Immunity
Regulation of Peptidyl Tyrosine Phosphorylation
Striated Muscle Cell Differentiation
Humoral Immune Response Mediated By Circulating Immunoglobulin
Inflammatory Response to Antigenic Stimulus
Negative Regulation of Leukocyte Cell Cell Adhesion
Response to Prostaglandin E
Regulation of Leukocyte Chemotaxis
Granulocyte Chemotaxis
Antigen Processing And Presentation Of Exogenous Peptide Antigen Via Mhc Class II
Tumor Necrosis Factor Superfamily Cytokine Production
Leukocyte Degranulation
Regulation of T Cell Migration
Positive Regulation of Natural Killer Cell Mediated Immunity
Negative Regulation of Cytokine Production
Smooth Muscle Cell Proliferation
NES
3
2
1
0
−1
−2
−3
Pathway size
100
200
300
400
B
Net Plot of GSEA KEGG
NFAT TF pathway
BCR 5 Pathway
Th1 Th2 Pathway
IL12 Pathway
TCR Pathway
IL2 STAT5 Pathway
NES
2
1
0
−1
−2
Pathway size
25
50
75
C
GSEA BIOCARTA
Running Enrichment Score
0.75
0.50
0.25
0.00
Term Name NES Pvalue
CTLA4 Pathway 2.45 < 0.001
Tob1 Pathway 2.26 < 0.001
NKT Pathway 2.26 < 0.001
Th1 Th2 Pathway 2.24 < 0.001
NO₂ IL12 Pathway 2.23 < 0.001
TCRα Pathway 2.19 < 0.001
ASBcell Pathway 2.15 < 0.001
CSK Pathway 2.13 < 0.001
Term Name NES Pvalue
T cytotoxic Pathway 2.13 < 0.001
T helper Pathway 2.12 < 0.001
Classic Pathway 2.09 < 0.001
IL12 Pathway 2.05 < 0.001
CTL Pathway 2.00 < 0.001
B lymphocyte Pathway 2.00 < 0.001
NK cells Pathway 1.92 0
Stathmin Pathway 1.86 < 0.001
TCR Pathway 1.86 < 0.001
Rank in Ordered Dataset
D
GSEA PID
Running Enrichment Score
0.5
0.0
−0.5
Term Name NES Pvalue
IL12 2pathway 2.53 < 0.001
CD8 TCR Pathway 2.49 < 0.001
CD8 TCR Downstream Pathway 2.49 < 0.001
TCR Pathway 2.41 < 0.001
IL12 STAT4 Pathway 2.36 < 0.001
IL4 2pathway 2.14 < 0.001
Term Name NES Pvalue
BCR 5pathway 1.96 < 0.001
TCR Calcium Pathway 1.95 0
IL2 STAT5 Pathway 1.92 < 0.001
NFAT TF pathway 1.89 < 0.001
IL27 Pathway 1.85 0
FcεRI Pathway 1.82 < 0.001
CXCR4 Pathway 1.82 < 0.001
IL2 1pathway 1.81 < 0.001
Term Name NES Pvalue
ALK2 Pathway −1.79 < 0.001
Rank in Ordered Dataset

Figure S6 | Construction of a scale-free co-expression network using WGCNA. (A) Sample dendrogram displaying hierarchical clustering of chordoma tissues (rows) based on their gene expression patterns. Each vertical branch line corresponds to a single sample, and the grouping patterns reflect global expression similarities. The heatmap below shows the assigned cluster memberships, with varying shades of red indicating distinct subgroups identified by dynamic tree cutting. **(B)** Plots depicting the selection of a soft-thresholding power (β) to achieve an approximate scale-free topology in the gene co-expression network. The top panel shows the scale-free topology model fit (R^2) for different β values; a horizontal red line indicates the selected threshold ($R^2 = 0.85$). The bottom panel displays mean connectivity (degree) as a function of the soft-threshold power. Increasing β reduces overall connectivity, ensuring that the resulting network exhibits scale-free properties. In this analysis, β=7 was selected as the optimal power setting.

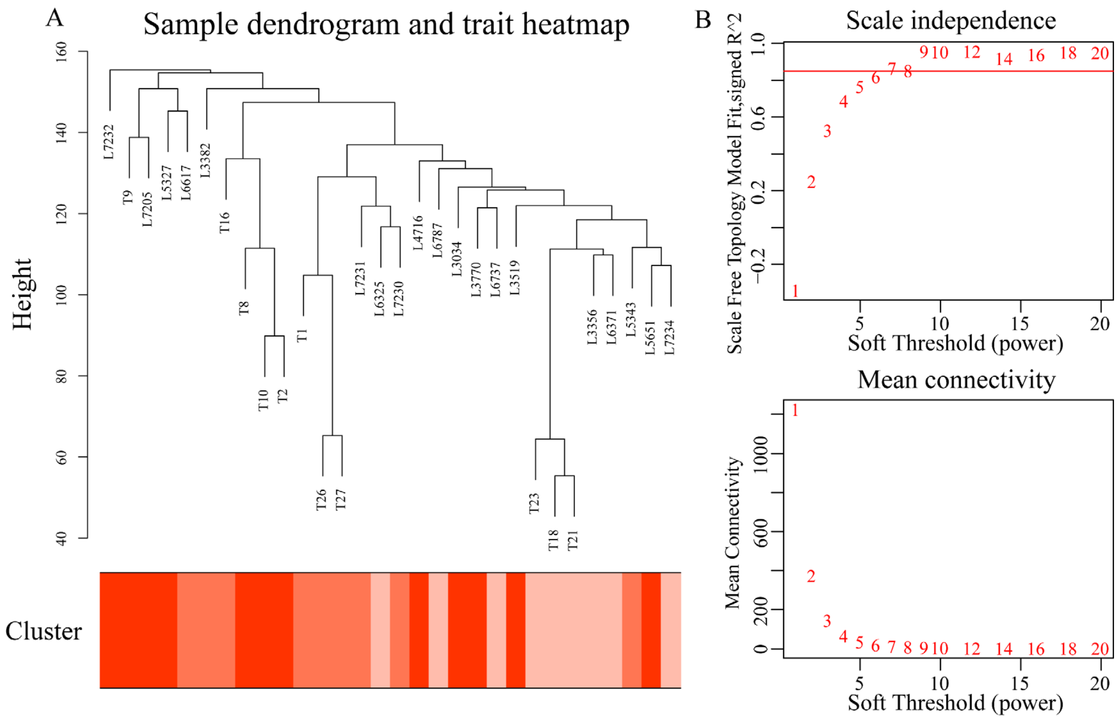

Table Legends

Table S1 | Comprehensive GSEA Results for Hallmark, C2, and C5 Gene Sets. Columns include pathway identifiers (ID, Description), gene set size (setSize), enrichment score (enrichmentScore), normalized enrichment score (NES), significance metrics (pvalue, p.adjust, qvalue), rank, leading_edge genes, and core_enrichment. These data outline the pathways enriched in chordoma versus normal tissues.

Table S2 | Immune Cell Infiltration and Immune-Related Analyses. This multi-sheet file provides outputs from different immune deconvolution algorithms (ABIS, CIBERSORT, EPIC, MCP-counter, quanTIseq, xCell), the ESTIMATE tool, and results for immune checkpoints, immune-related genes, and immunophenocore scoring. Each sheet shows infiltration levels or gene-set scores for chordoma samples.

Table S3 | Immune Checkpoints and Immune Gene Distributions Across Chordoma Samples. Rows (e.g., T18, T1, T21, T23, T26, T27, T9) represent individual chordoma samples or subgroups, with columns listing expression or abundance values for various immune checkpoints and immune-related genes, highlighting differences across samples.

Table S4 | Enrichment Analysis of Cluster-Specific DEGs (C2 and C5 Databases). Columns include ID, Description, setSize, enrichmentScore, NES, pvalue, p.adjust, qvalue, rank, leading_edge, core_enrichment, Database, and status. These metrics reflect the significance of enriched pathways derived from cluster-level differential expression in chordoma.

Table S5 | GO and KEGG Functional Annotation of Core T Cell-Related Genes.

Columns (Description, GeneRatio, BgRatio, pvalue, p.adjust, qvalue, geneID, Count) provide key statistics for enriched Gene Ontology (GO) terms and KEGG pathways linked to T cell function. This table highlights the biological processes in which identified hub genes are involved.
